# The Effect of the Great Recession on Italian Life Expectancy

**DOI:** 10.1007/s11113-023-09755-5

**Published:** 2023-01-28

**Authors:** Giambattista Salinari, Federico Benassi, Gianni Carboni

**Affiliations:** 1 Department of Economics and Business, University of Sassari, Sassari, Italy; 2 Department of Political Sciences, University of Naples Federico II, Naples, Italy

**Keywords:** Life expectancy, Economic crisis, Italy, Artificial control, Health care expenditure, Causal inference

## Abstract

The 2008 economic crisis, also called the Great Recession, produced only a moderate rise in unemployment in Italy, but the consequences for public debt management were far more serious. Italy makes for a good case study for evaluating the effect on life expectancy at birth of the cost containment program in the health care system, implemented after the crisis began. To this end we employed the Artificial Control method using the data from the Human Mortality Database to assess the causal effect of the 2008 economic crisis on the subsequent evolution of life expectancy at birth (until 2019, before the onset of the COVID-19 pandemic). Our analysis identifies a significant deceleration in the progression of Italian life expectancy. Ten years after the onset of the crisis, Italy appears to have lost almost 1 year of life expectancy with respect to what would have been expected had the crisis never happened.

## Introduction

When long time series on the evolution of mortality first became available it emerged that, over the last century and half, life expectancy at birth had followed, in Western countries, approximately a linear trend with a steady upward slope (Oeppen & Vaupel, 2002; White, 2002). This discovery has subsequently been used to counter the idea of a biological limit on human life, because if such a limit existed, we should have seen at a certain point a deceleration in the progression of life expectancy. A study by White (2002) showed, by contrast, that at least in the period 1955–1995, life expectancy progression displayed a slight tendency to acceleration. This study estimated that the overall progression in life expectancy led to a gain of about 20–25 years over a century.

This positive picture of sustained, constant, and generalized progression in life expectancy was, at the time, only contradicted by events in two western countries: the USA and Denmark. Since the 1980s life expectancy decelerated significantly in both countries and the two were at the bottom of the classification of OECD countries in 2000s. The two countries then had different fates. Denmark started to catch up, at least in part, with other OECD countries (Christensen et al., 2010), whereas the situation in the USA, because of the opioid crisis, actually worsened (Case & Deaton, 2020; Wilmoth et al., 2010).

With the 2008 financial crisis (the Great Recession) the number of countries that started to deviate from the secular linear trend of life expectancy grew. In the UK the period after the crisis saw a substantial stall in life expectancy with practically no significant progression over a decade (Murphy et al., 2019; Raleigh, 2018). Belgium, France, and Germany also showed some signs of deceleration in life expectancy, although not so strong as those identified in the UK (Murphy et al., 2019). The reasons for this recent trend are largely unknown and only a few hypotheses have so far been advanced to explain this change (Murphy et al., 2019): (1) Austerity policies that in the aftermath of the crisis led to programs of cost reduction that particularly affected the health sector; (2) The reduction in the improvement in cardiovascular mortality and other causes of death; (3) The rise in the burden of transmittable diseases; and (4) Tempo effects.

This picture, however, is further complicated by the fact that since 2008 many European countries have experienced a marked reduction in mortality (Ballester et al., 2019; Baumbach & Gulis, 2014; Cervini-Plá & Vall-Castelló, 2021; Regidor et al., 2016; Tapia Granados & Ionides, 2017; Toffolutti & Suhrcke, 2014). This appears, perhaps surprisingly, to be especially true for those countries in which the rise in unemployment rate was particularly intense: Spain, Ireland, Slovenia, and the Baltic States (Salinari & Benassi, 2022).

In summary, after the Great Recession mortality followed in Europe a somewhat paradoxical evolution. The few countries that suffered a relatively mild form of crisis, such as the UK, showed a marked deceleration in life expectancy progression. Yet other countries, which suffered a more intense crash, showed a marked mortality reduction.

With this paper we want to contribute to this debate by analyzing the case of Italy trough methods borrowed from causal inference.

## The Great Recession in Italy

**Fig. 1:**
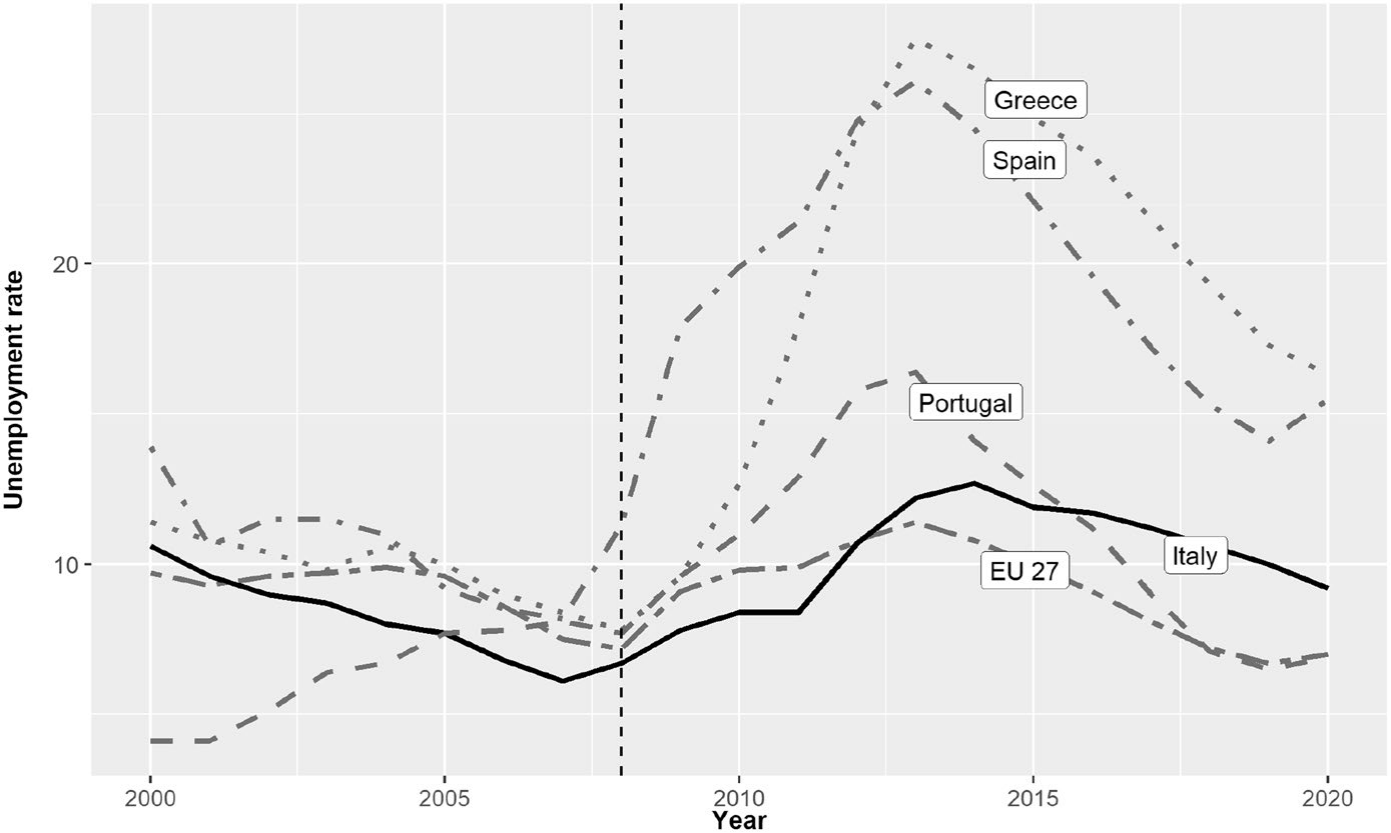
Unemployment rate (percentage of the population in the labor force). 2000–2020. *Source* Our elaboration on Eurostat data (Eurostat data browser)

In Italy the Great Economic Recession of 2008 proved quite different from the crisis in other southern European countries, like Greece, Spain, and Portugal. The increase in unemployment was relatively small (Fig. 1), while the financial effects of the crisis proved strong. These led to a program of cost reductions in the public sector which would have knock on effects through the following decade.

Health care was particularly affected by cuts. This has caused controversy in the context of the Covid-19 pandemic. Indeed, it has been said that Italy was hit particularly hard by the pandemic because of the reduction in healthcare spending in the previous years (Armocida et al., 2020; Egidi & Manfredi, 2021; Ricci et al., 2020). For this reason, at the beginning of the pandemic, in March 2020, the Italian National Institute of Statistics (Istat) released a document for the Italian Parliament which addressed, among other things, cost containment in health care.^1^ During the last decades in Italy, with the aim of containing and even reducing public debt, public health spending was reduced significantly (Cartabellotta et al., 2019). Between 2010 and 2017 the National Health Service (NHS) registered a reduction of 42,861 units (− 6.7%): the number of doctors decreased by − 5.9% and the number of nurses by − 6.7%. The reduction affected not only the medical staff but also health facilities. Indeed, from 2010 to 2018, the number of beds in hospitals fell by an annual average of 1.8%, continuing a trend underway since the mid-1990s (Istat, 2020).

**Fig. 2:**
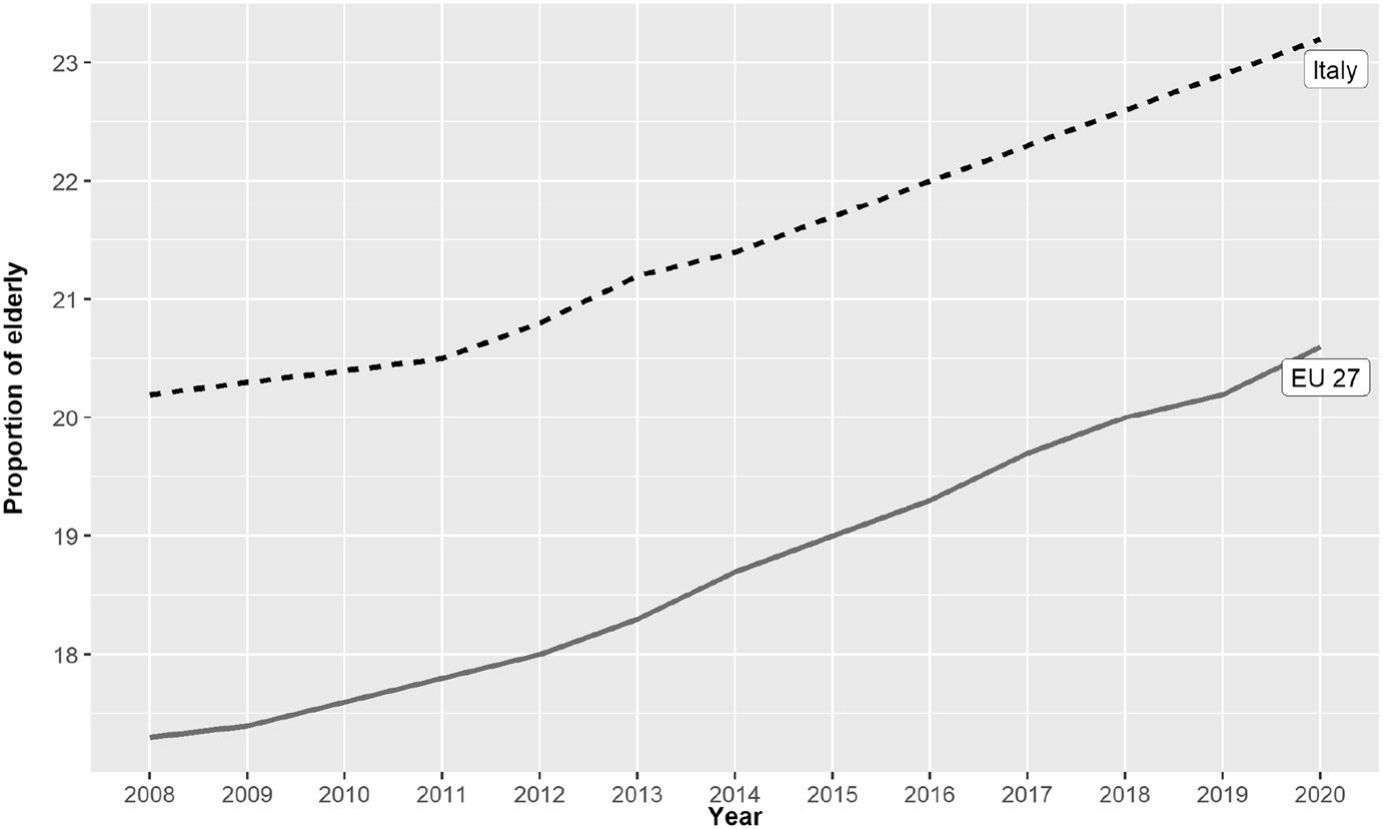
Proportion of population aged 65 or older (percentage of total population). Italy 2009–2020. *Source* Our elaboration on Eurostat data (Eurostat data browser)

Conversely, the potential demand for health services has grown over the same period and quite strongly too. Figure 2 shows the proportion of elderly people in Italy from 2009 to 2020. This is a proxy for the potential demand on health care since older Italians typically have the greatest need for health care (Payne et al., 2007). The growth is significant (from 20.3% in 2009 to 23.2% in 2020) and the proportion of the elderly in Italy is significantly higher than the European average (EU 27): 17.4% in 2009 and 20.6% in 2020.

Therefore, if we want to read this dynamic in economic terms, potential demand has increased, while real supply has decreased. This dual dynamic has entailed, we would suggest, some long-term effects on the dynamics of life expectancy. To study the effects of the Great Recession in Italy, we used the Artificial Control method (Carvalho et al., 2018). Based on the evolution of life expectancy in those countries that have been less affected by the crisis, we have reconstructed a counterfactual evolution of life expectancy for a “version” of Italy in which the Great Recession never took place.

## Data and Methods

According to the potential outcome framework (Rubin, 1974) the causal effect of an intervention is defined as the contrast between two counterfactual outcomes. This implies that to assess the effect of the crisis on life expectancy, one should estimate what would have been the evolution of Italian life expectancy had the Great Recession never happened.

A possible solution here consists in comparing the evolution of life expectancy in Italy (*the treated unit*) with that of a country “similar” to Italy, but one where no crisis took place (*the control unit*). The weakness of this approach lies in the fact that it entails some degree of arbitrariness in the selection of the control unit. To solve this problem, Abadie (2021) offered an ingenious solution. Information would be collected on several different potential control units or peers, which taken together go to make up the donor pool. By combining the peers’ characteristics together, we can then obtain a *synthetic control* as similar as possible to the treated unit. This combination of the characteristics of the peers is accomplished by attributing different weights to said peers. The synthetic control is eventually computed as a weighted average of the peers in the donor pool.^2^ In the present context we use a variant of the Synthetic Control (SC) methodology called Artificial Control (AC).

The AC method (Carvalho et al., 2018) presents several advantages with respect to the SC method.

First, SC relies on a convex combination of control units. This means that the weights attributed to the control units must be positive and sum to 1. Because of this limitation it is not possible to apply the SC method when the treated unit has a value (life expectancy) that, for instance, exceeds those observed for the peer in the donor pool. More importantly, the fact of relying on a convex combination induces a bias in the estimator (Ferman & Pinto, 2016). The AC method has the advantage of not requiring a convex combination and negative weights can, thus, be employed.

Second, the weights in the SC method are estimated by taking the averages over time of the observed variables for each control unit. In this manner, the temporal dynamic of the process is lost and the weights are estimated using pure cross-sectional data. The AC method, instead, allows for the preservation of the time dimension of the data.

Third, and perhaps more importantly, in the SC method there is not a formal inferential procedure for hypothesis testing: inference is performed by means of a complex permutation test (Abadie, 2021; Abadie et al., 2010). In the case of the AC method a relatively simple parametric test based, instead, on the chi-square distribution can be performed. This last point particularly led us to use the AC method in the present context.

As the SC method the application of the AC method is based on two key assumptions (Fonseca et al., 2018):

1. The control units are not affected by the intervention (the crisis) and
2. The data are trend stationary.

In the formation of the donor pool, we excluded non-European countries that suffered a mild form of crisis (such as Australia, Canada, and Japan), because we considered them too different from Italy in terms of the organization of their Health Care System and more in general, in terms of lifestyle. Following the method set out in the paper of Tapia Granados and Ionides (2017), we first identified our donor pool, namely nine European countries where the crisis had only mild effects (or no effect): the variation in the rate of unemployment was less than 2 percentage points between 2007 and 2010. We selected eight of the nine with a life expectancy at birth series available in the Human Mortality Database (HMD) from 1970 to 2019: Austria, Belgium, Finland, France, West Germany, the Netherlands, Norway, and Switzerland. This period was selected for three main reasons. First, we wanted to stop the analysis just before the onset of the COVID-19 pandemic. Second, the 1970–2007 (pre-treatment) period is, in terms of life expectancy progression, rather homogeneous across the European countries here considered. It is characterized by a rapid rise in life expectancy thanks, above all, to a significant reduction in cardiovascular mortality (the so-called cardiovascular revolution). Third, Fonseca et al. (2018) suggest using at least 40–50 observations when applying the AC method. However, since our results may depend on the length of the time series employed in the procedure, we resolved to repeat our tests by also using the shorter period 1990–2019.

We then differentiated these series, because life expectancy is not stationary (the series are generally integrated of order 1; see below for a verification of this statement). Indeed, it has been shown that techniques such as SC and AC may yield biased results if the original series are not stationary (Carvalho et al., 2016). We finally assume that the intervention, the crisis, started in 2008 (${T}_{0}$) and lasted until the end of our series in 2019 ($T$).

To build the artificial control, we first ran the following linear model over the pre-intervention period 1971–2007:

$$\Delta y_{t} = \alpha + \sum\nolimits_{i = 1}^{p} {\beta_{i} \Delta x_{i,t} } + \varepsilon_{t} ,\quad t = 1, \ldots ,T_{0} - 1,$$

where $\Delta {y}_{t}={y}_{t}-{y}_{t-1}$ is the change in life expectancy in Italy between $t$ and $t-1$, ${\Delta x}_{i,t}={x}_{i,t}-{x}_{i,t-1}$ is the change in life expectancy in control unit $i$ between year $t$ and $t-1$, $p$ is the number (eight) of countries taken as control units, $\alpha$ and ${\beta }_{i}$ are the model parameters, and ${\varepsilon }_{t}$ is the error term. The model can also include lagged regressors (${\Delta x}_{i,t-1}$), but since the inclusion of these additional terms did not yield a significant improvement in model accuracy, we preferred the simpler version without lags. The model parameters can be, in principle, estimated via ordinary least squares (OLS), but the AC method works better if LASSO regression is employed. This means that instead of minimizing the following quantity with respect to the parameters $\alpha$ and ${\beta }_{i}$:

$${\text{SSE}} = \sum\nolimits_{t = 1}^{{T_{0} - 1}} {\left( {\Delta y_{t} - \alpha - \sum\nolimits_{i = 1}^{p} {\beta_{i} \Delta x_{i,t} } } \right)}^{2} ,$$

we minimize the quantity:

$${\text{SSE}} + \lambda \sum\nolimits_{i = 1}^{p} {\left| {\beta_{i} } \right|} ,$$

where $\lambda$ is a tuning parameter to be determined through cross-validation. For elevated values of $\lambda$ it can be shown that the LASSO method forces some (or even all) of the ${\beta }_{i}$ parameters to be exactly zero. So, the LASSO method can be viewed as a way to select only those control units that are closer to the treated unit in terms of their life expectancy evolution. The variance–covariance matrix of the model was estimated through the Newey and West (1987) sandwich estimator so as to take into account both heteroskedasticity and autocorrelation.

The coefficients $\widehat{\alpha }$ and ${\widehat{\beta }}_{i}$ estimated on the pre-treatment period 1971–2007 can then be employed to predict the variation in life expectancy $\Delta {\widehat{y}}_{t}$ over the post-treatment period 2008–2019 by combining the post-treatment series of the countries in the donor pool. By contrasting the actual series ($\Delta {y}_{{T}_{0}, \ldots,}\Delta {y}_{T}$) with the predicted series ($\Delta {\widehat{y}}_{{T}_{0}, \ldots,}\Delta {\widehat{y}}_{T}$) for the post-treatment period we get the average effect $\delta$ of the crisis on the evolution of life expectancy:

$$\delta = \frac{1}{{T - T_{0} + 1}}\sum\nolimits_{{i = T_{0} }}^{T} {\left( {\Delta y_{i} - \Delta \hat{y}_{i} } \right).}$$

It is important to note that we cannot exclude the possibility that at least some of the donor pool countries suffered a mild form of recession. We will, therefore, consider in the present context the estimates of $\delta$ as a lower bound for the true causal effect of interest. Carvalho et al. (2018) shows that $\delta$ is asymptotically normally distributed. This result may be used to build a confidence interval for $\delta$:

$$\delta \pm \frac{{s_{\varepsilon } }}{\sqrt T }z_{\alpha /2} ,$$

where ${s}_{\varepsilon }$ is the standard deviation of the residuals of our regression model and ${z}_{\alpha /2}$ is the quantile of the normal distribution that leaves in the right tail a probability of $\alpha /2$. The formal test for the null hypothesis that $\delta =0$ is instead performed by computing the quantity

$$w = T\left( {\delta s_{\varepsilon } } \right)^{2} ,$$

which asymptotically conforms to a chi-square distribution with one degree of freedom. To perform all the steps of this procedure we used the ArCo R package (Fonseca et al., 2018).

As a final step, we repeatedly applied the AC procedure to all countries in our donor pool to produce a series of placebo tests. According to our hypotheses, we thus expect to identify, after the crisis onset, a stronger deceleration in life expectancy when the country analyzed is Italy (the main test); by contrast we expect to find a weaker deceleration, or no deceleration at all, when the procedure is applied to the other donor pool countries which were comparatively less affected by the crisis (placebo tests).

## Quantifying the Effects of the Crisis on Life Expectancy

**Fig. 3:**
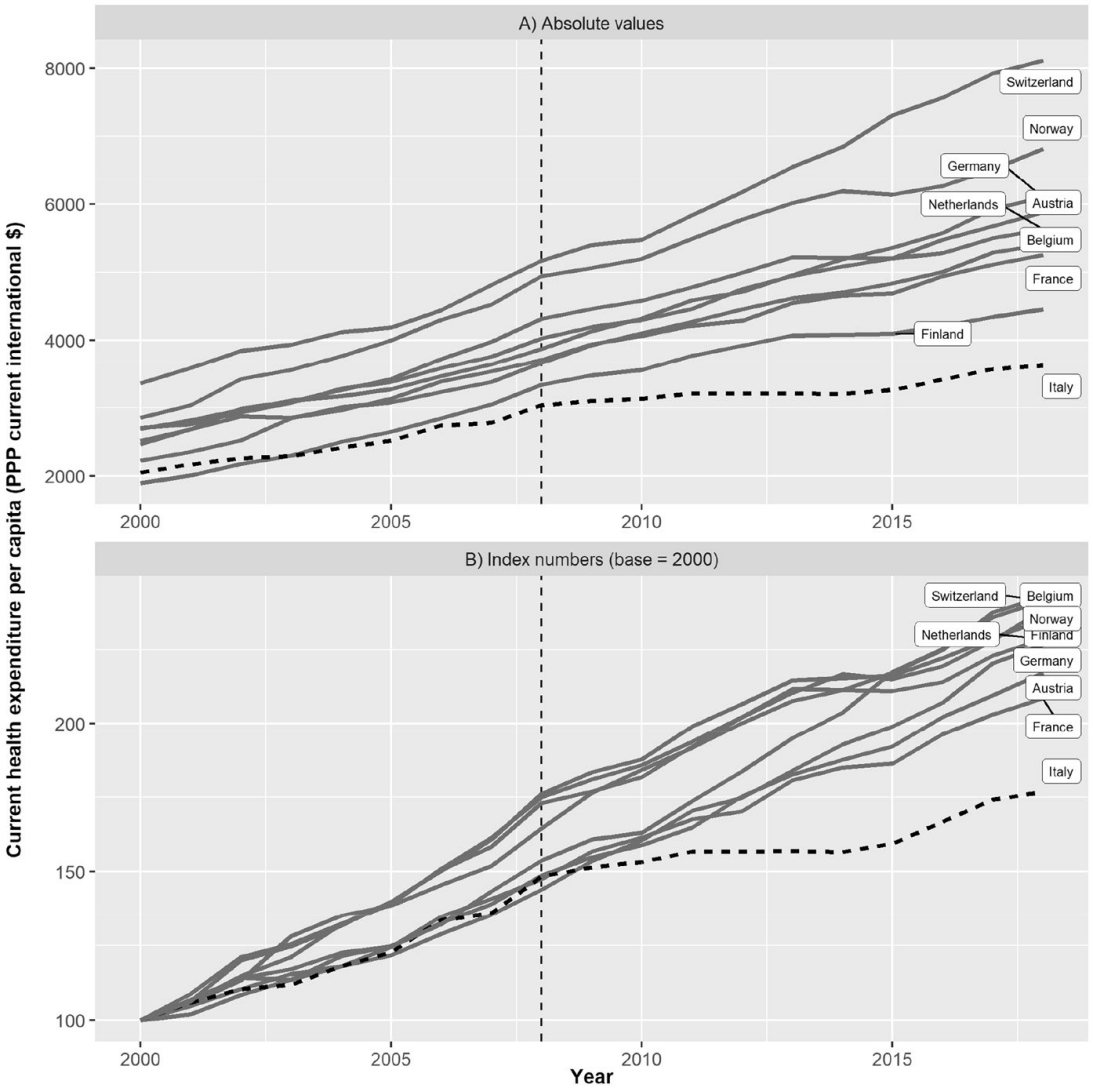
Current health expenditure in selected European countries over the period 2000–2018. Panel A shows absolute values of per capita current health expenditure. Panel B shows index numbers with the year 2000 as point of reference. *Source* Our elaboration on World Bank data

Following the classification proposed by Tapias Granados and Ionides (2017) Italy assumes an intermediate position between our control group—where the crisis produced almost no effect on unemployment—and countries, such as Spain and Greece, where the increase in unemployment was steepest. The rise in unemployment, however, does not exhaust all effects entailed by the crisis. In some countries the crisis also brought in the next years a deceleration in health expenditure. This is exactly what happened in Italy. Figure 3 shows the evolution of *per capita current health expenditure* during the period 2000–2019 for Italy and the control units. Specifically, we show in Fig. 3A the absolute values of this indicator in current US dollar, whereas in Fig. 3B we computed index numbers with the year 2000 as our point of reference. From these figures it emerges that at the beginning of the crisis Italy was already spending less on health care than the control units (Fig. 3A) and that from the onset of the crisis a strong divergence took place between Italy and the control units (Fig. 3B). The divergence appears particularly marked during the period 2008–2015.

The crisis, thus, induced multiple changes (in unemployment, health expenditure, etc.) in Italy in comparison to the control units. The important point here is that through the Artificial Control method we will measure the joint effect on life expectancy produced by all these different changes.

To determine the order of integration of life expectancy series we ran two kinds of test for each country: we first employed the KPSS test (Kwiatkowski et al., 1992) on the levels of our series to determine whether they are non-stationary and then we applied the Phillips–Perron test (Perron, 1988) on the differentiated series to determine whether they are stationary. The difference between these two kinds of test is that in the KPSS test the null hypothesis is represented by the condition of stationarity, whereas in the Philips–Perron test the null is represented by non-stationarity. In both series of tests, we thus set a null hypothesis which represents the opposite of what we want to prove. Both kinds of test allow for heteroscedasticity in the series and for this reason they have been preferred to the ADF test (Said & Dickey, 1984).

The results of the KPSS tests (Table 1, first column) reject the condition of stationarity for all series except two: Finland and Switzerland. The results of the Philips–Perron (Table 1 second column) show that the first differences of life expectancy are stationary in all countries. Since stationarity is a required condition for applying the SC and AC methods, we resolved to use the differentiated series for our analysis.

**Table 1:** Order of integration of the series and the coefficients of LASSO regression

Country	KPSS test *p* value	PP test *p* value	Model coeff
Intercept	–	–	0.0665
Austria	< 0.01	< 0.01	–
Belgium	< 0.01	< 0.01	–
Finland	> 0.10	< 0.01	− 0.1077
France	0.02	< 0.01	0.7045
Germany (West)	< 0.01	< 0.01	–
Italy	< 0.01	< 0.01	–
The Netherlands	0.02	< 0.01	− 0.166
Norway	< 0.01	< 0.01	–
Switzerland	> 0.10	< 0.01	0.3357

*Source* Our elaboration on HMD data

We then ran a LASSO regression in which the variations in Italian life expectancy represent the response variable, whereas the variations in life expectancy observed in the countries of the donor pool represent potential explanatory variables. Through cross-validation we estimated $\lambda =0.01783$, then the LASSO regression selected four countries in the donor pool for the analysis: Finland, France, the Netherlands, and Switzerland. The estimated coefficients (see the last column in Table 1) indicate how the variations observed in these four countries must be combined to form the artificial control. The weights are greatest for France (0.70) and Switzerland (0.33), two countries bordering Italy. To give you an idea of the similarity of these countries in terms of life expectancy, consider that in 2007, just before the onset of the crisis, life expectancy was 80.97 in France, 81.50 in Italy, and 81.7 in Switzerland.

**Fig. 4:**
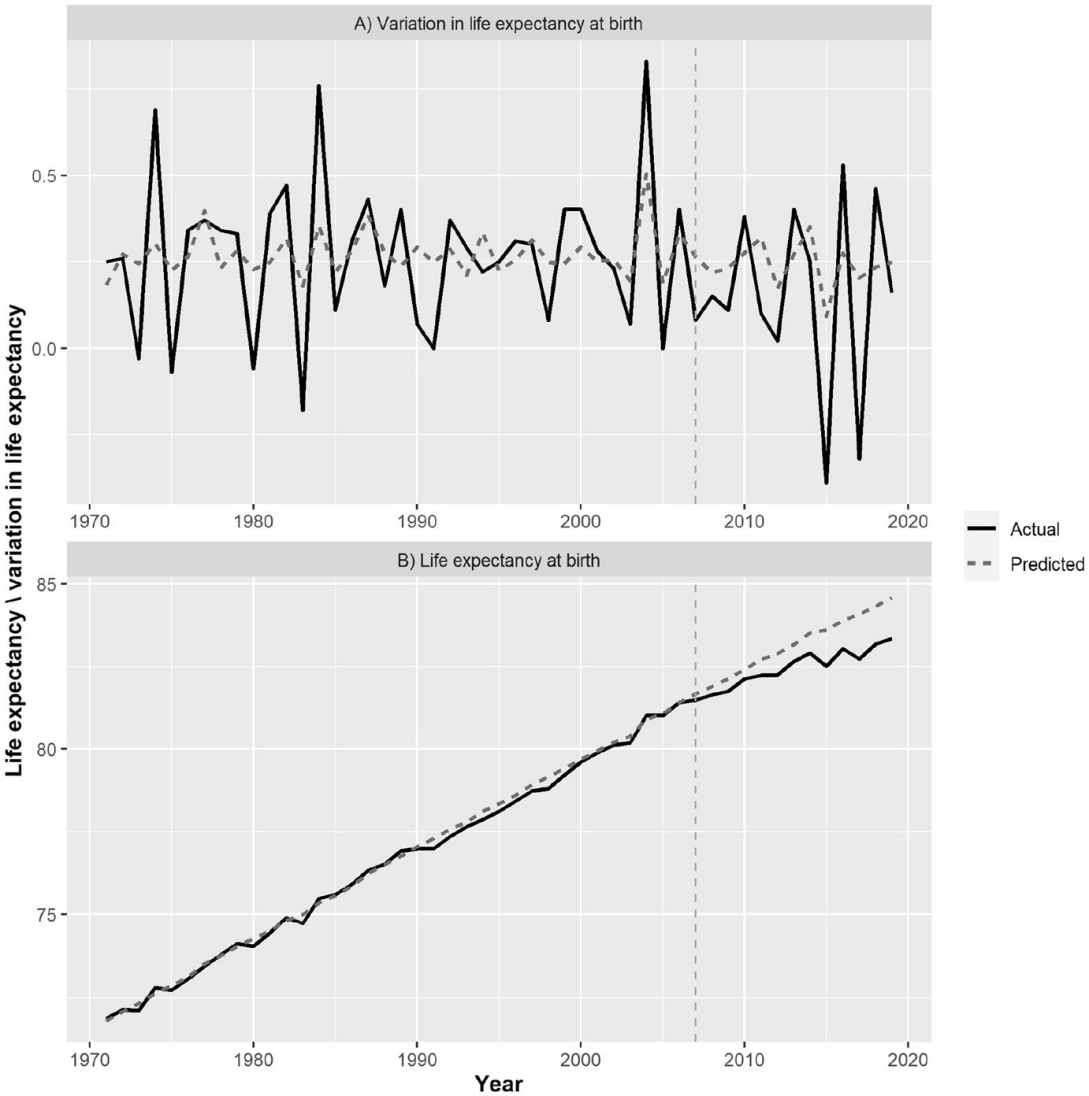
The evolution of life expectancy at birth in Italy 1971–2018. Panel A shows actual and predicted annual variations in life expectancy. Panel B reports actual and predicted annual levels of life expectancy. *Source* Our elaboration on HMD data

The main results of our analysis are to be found in Fig. 4, where panel A shows the actual ($\Delta {y}_{t}$) and the predicted ($\Delta {\widehat{y}}_{t}$) series of the annual *variation* in life expectancy, whereas panel B reports the evolution of actual (${y}_{t}$) and predicted (${\widehat{y}}_{t}$) *levels* of life expectancy. To compute the predicted life expectancy series, we simply added to the value of life expectancy recorded in 1970 the cumulated series of predicted variations observed thereafter.

What interests us most here is the differential between the actual and the predicted (artificial) series that materializes after the onset of the economic crisis (marked by a vertical dashed line). In Fig. 4A the actual series tends to lie below the counterfactual series after the crisis. For this reason, the estimate of $\delta$—the average difference between the two series during the post-treatment period—leads to a negative value of − 0.094 (95% confidence interval: − 0.153, − 0.035). This indicates that after the onset of the crisis the evolution of life expectancy in Italy has progressed at a significantly (*p* value = 0.002) slower pace than we would have expected had the crisis not happened.

This is easier to see if we look at life expectancy levels. During the pre-intervention period life expectancy in Italy appears to follow an almost perfect linear trend with a slope of about 0.27. This is in line with what is known about the evolution of life expectancy at birth in other contexts (Lee, 2019; Oeppen & Vaupel, 2002) for which a slope of about 0.3 was estimated. The beginning of the crisis, however, clearly represents a turning point in the recent history of Italy. The estimate of − 0.094 for $\delta$ corresponds to a deceleration in the progression of life expectancy of about 30% with respect to the pre-crisis value. So, if before the crisis there was a gain in life expectancy of 3 years every 10 years, after the crisis this fell to a gain of only 2 years. Figure 4B shows that in the first decade after the crisis began, Italy lost about 1 year of life expectancy with respect to what would have been expected based on its previous dynamics. To check the robustness of our results we repeated our analysis by focusing this time on the shorter period 1990–2019 and obtained similar results: the difference between the actual and the counterfactual series in the post-treatment period ($\delta$) was equal to 0.079, with a 95% confidence interval of (− 014, − 0.13) and a *p* value of 0.019. When, however, the analysis was restricted to the remaining life expectancy at age 65, the effect of the crisis proved to be not significant (results not shown). This seems to indicate that the deceleration in life expectancy at birth observed after the crisis is not due to worsening conditions among the elderly population. The placebo tests performed on the countries in the donor pool did not find a significant effect of the crisis in six out of eight countries; in two countries, West Germany and Austria, the crisis had a similar effect to that observed in Italy, although with a smaller magnitude. Since the LASSO regression (see Table 1) excluded these two countries from the composition of the artificial control, this does not represent an issue for our previous analysis.

## Discussion and Conclusion

The Italian economic crisis saw policies tailored to the containment of public debt and only moderate rises in unemployment levels. In this context our analysis identified a deceleration in Italian life expectancy at birth which coincides with a phase of stagnating current health expenditure.

The pre-crisis period 1970–2007 was characterized, instead, by a steady progression in life expectancy pushed by a significant reduction in cardiovascular mortality. This surprising stability can be checked by looking at Fig. 4B where it is shown that for this entire period life expectancy progression stays very close to a straight line. The relevance of the break that manifest in 2008 can be, in our view, fully understood only if we compare the evolution of life expectancy after 2008, with the great stability that instead characterized the progression of life expectancy before this epoch.

Italy seems, in this respect, to share a similar fate to European countries such as Austria, Belgium, Germany, and the UK which experienced, after 2007, a relatively moderate rise in unemployment, followed by a significant deceleration in life expectancy. However, the specific mechanisms through which the economic crisis and life expectancy deceleration interacted in the Italian context remain largely unknown. The analysis of the evolution of remaining life expectancy at age 65 did not identify a significant deceleration process. According to this analysis the elderly population appears to have been spared by the crisis. A possibility is that during the reduction in healthcare spending the Italian health care system concentrated its resources mainly on the frailest segment of the population, leaving the other segments somewhat unsheltered. Whatever the right explanation, it should be noticed that several contexts exist in which a deceleration in life expectancy progression took place because of the worsening health conditions among relatively younger parts of the population. In the USA, for instance, Case and Deaton (2020) have shown that the recent dynamic of life expectancy have been heavily affected by the rise in mortality of a very specific group: low educated white men in the 45–54 age bracket. In Greece, instead, the economic crisis coincided with a phase of increasing infant mortality and reduced cardiovascular mortality (Filippidis et al., 2017).

For those European countries in which unemployment rose significantly after the crisis the evidence collected points in the other direction: the worsening of economic conditions induced a faster decrease in mortality and thus a faster increase in life expectancy. The reason for this counterintuitive change seems to be found in two main mechanisms: (1) Crises make the environment temporarily healthier by reducing air pollution, work and car accidents, and the spread of transmittable diseases (because of reduced work and commercial interactions) and (2) Crises induce people to abandon unhealthy habits such as smoking and drinking and to increase the time they devote to physical activity, sport, and medical care (Catalano et al., 2011; Ruhm, 2000, 2016; Tapia Granados, 2005).

Thus, one of the key variables for explaining the dynamics of life expectancy in Italy seems to be represented by unemployment. Let’s compare, for instance, Italy to Greece and Spain. Even though Greece and Spain went through a phase of stagnation/contraction in *per capita* health expenditure, as Italy did, they did not see a deceleration in life expectancy at birth (Filippidis et al., 2017; Kentikelenis et al., 2014; Regidor et al., 2013, 2016). It must be remembered, however, that in these countries the crisis also produced a huge rise in unemployment: levels reached well over 25% of the working population (see Fig. 1). So, it is possible that in Spain and Greece the negative effect due to the contraction in *per capita* health expenditure was counterbalanced by the positive (for life expectancy) effect due to rising unemployment levels. By contrast, in Italy, where the rise in unemployment was moderate at the national level (specific sub-national contexts may have been, instead, characterized by elevated unemployment levels) the effect of the stagnation in current health expenditure eventually prevailed and there was a deceleration in life expectancy progression.

Italy’s experience is important, we would suggest, because it indicates that there may be, besides the two main mechanisms connected to the increase in unemployment, other mechanisms through which crises influence the evolution of mortality. Importantly, one of the effects of the increase in unemployment may be to hide these other mechanisms. For this reason, one may derive the superficial impression, while looking at Spain or Greece that in these countries the cuts to the health care system did not produce significant effects on the subsequent evolution of life expectancy. Italy seems to indicate the opposite that life expectancy may be extremely sensitive to cuts in the health care systems. This is a point that should be kept in mind, along with other economic goals, when planning health care reforms.

## Footnotes

1 https://www.istat.it/it/archivio/240199.

2 See for recent applications of this method: Azzolini and Guetto (2017), Bonneau et al. (2022), Geloso and Pavlik (2020), Grier and Maynard (2016).

## References

[CR1] Abadie A (2021). Using synthetic controls: Feasibility, data requirements, and methodological aspects. Journal of Economic Literature.

[CR2] Abadie A, Diamond A, Jens H (2010). Synthetic control methods for comparative case studies: Estimating the effect of California’s Tobacco Control Program. Journal of the American Statistical Association.

[CR3] Armocida B, Formenti B, Ussai S, Palestra F, Missoni E (2020). The Italian health system and the COVID-19 challenge. The Lancet Public Health.

[CR4] Azzolini D, Guetto R (2017). The impact of citizenship on intermarriage: Quasi-experimental evidence from two European Union Eastern enlargements. Demographic Research.

[CR5] Ballester J, Robine J-M, Herrmann FR, Rodó X (2019). Effect of the Great Recession on regional mortality trends in Europe. Nature Communications.

[CR6] Baumbach A, Gulis G (2014). Impact of financial crisis on selected health outcomes in Europe. European Journal of Public Health.

[CR7] Bonneau DD, Hall JC, Zhou Y (2022). Institutional implant and economic stagnation: A counterfactual study of Somalia. Public Choice.

[CR8] Cartabellotta, N., Cottafava, E., Luceri, R., & Mosti, M. (2019). Il definanziamento 2010–2019 del servizio sanitario nazionale. Report Osservatorio GIMBE n. 7/2019.

[CR9] Carvalho, C. V., Masini, R. P., & Medeiros, M. C. (2016) *The perils of counterfactual analysis with integrated processes*. Working paper, Pontifical Catholic University of Rio de Janeiro (pp. 92–96). SSRN. https://ssrn.com/abstract=2894065 or 10.2139/ssrn.2894065

[CR10] Carvalho CV, Masini RP, Medeiros MC (2018). ArCo: An artificial counterfactual approach for high-dimensional panel time-series data. Journal of Econometrics.

[CR11] Case A, Deaton A (2020). Deaths of despair and the future of capitalism.

[CR12] Catalano R, Goldman-Mellor S, Saxton K, Margerison-Zilko C, Subbaraman M, LeWinn K, Anderson E (2011). The health effects of economic decline. Annual Review of Public Health.

[CR13] Cervini-Plá M, Vall-Castelló J (2021). Business cycle and mortality in Spain. The European Journal of Health Economics.

[CR14] Christensen K, Davidsen M, Juel K, Mortensen L, Rau R, Vaupel JW, Crimmins EM, Preston SH, Cohen B (2010). Trends in Denmark and Sweden and some potential explanations. International differences in mortality at older ages. Dimensions and sources.

[CR15] Egidi V, Manfredi P (2021). Population dynamics and demography of Covid-19. Introduction. Genus.

[CR16] Ferman, B., & Pinto, C. (2016). *Revisiting the synthetic control estimator*. Working paper, São Paulo School of Economics.

[CR17] Filippidis FT, Gerovasili V, Millett C, Tountas Y (2017). Medium-term impact of the economic crisis on mortality, health-related behaviours and access to healthcare in Greece. Scientific Reports.

[CR18] Fonseca YR, Masini RP, Medeiros MC, Vasconcelos GFR (2018). ArCo: An R package to Estimate Artificial Counterfactuals. The R Journal.

[CR19] Geloso V, Pavlik JB (2020). The Cuban revolution and infant mortality: A synthetic control approach. Explorations in Economic History.

[CR20] Grier K, Maynard N (2016). The economic consequences of Hugo Chavez: A synthetic control analysis. Journal of Economic Behavior & Organization.

[CR21] Istat. (2020). Esame del disegno di legge A.S. 1766. Conversione in legge del decreto-legge 17 marzo 2020, n.18. Memoria scritta dell’Istituto Nazionale di Statistica. 5a Commissione programmazione economica e bilancio Senato della Repubblica. Roma, 26 Marzo 2020. https://www.istat.it/it/archivio/240199

[CR22] Kentikelenis A, Karanikolos M, Reeves A, McKee M, Stuckler D (2014). Greece’s health crisis: From austerity to denialism. The Lancet.

[CR23] Kwiatkowski D, Phillips PCB, Schmidt P, Shin Y (1992). Testing the null hypothesis of stationarity against the alternative of a unit root. Journal of Econometrics.

[CR24] Lee R, Bengtsson T, Keilman N (2019). Mortality forecasts and linear life expectancy trends. Old and new perspectives on mortality forecasting.

[CR25] Murphy, M., Luy, M., & Torrisi, O. (2019). *Stalling of mortality in the United Kingdom and Europe: An analytical review of the evidence*. London School of Economics, Working Paper Series November 2019.

[CR26] Newey W, West K (1987). A simple, positive semi-definite, heteroskedasticity and autocorrelation consistent covariance matrix. Econometrica.

[CR27] Oeppen J, Vaupel JW (2002). Broken limits to life expectancy. Science.

[CR28] Payne G, Laporte A, Deber R, Coyte PC (2007). Counting backward to health care’s future: Using time-to-death modeling to identify changes in end-of-life morbidity and the impact of aging on health care expenditures. Milbank Quarterly.

[CR29] Perron P (1988). Trends and random walks in macroeconomic time series. Journal of Economic Dynamics and Control.

[CR30] Raleigh VS (2018). Stalling life expectancy in the UK. We must look at austerity and beyond for underlying causes. BMJ.

[CR31] Regidor E, Barrio G, Bravo MJ, de la Fuente L (2013). Has health in Spain been declining since the economic crisis?. Journal of Epidemiology and Community Health.

[CR32] Regidor E, Vallejo F, Tapia Granados JA, Viciana-Fernández FJ, de la Fuente L, Barrio G (2016). Mortality decreases according to socioeconomic groups during the economic crisis in Spain: A cohort study of 36 million people. The Lancet.

[CR33] Ricci G, Pallotta G, Sirignano A, Amenta F, Nittari G (2020). Consequences of COVID-19 outbreak in Italy: Medical responsibilities and governmental measures. Frontiers in Public Health.

[CR34] Rubin D (1974). Estimating causal effects of treatments in randomized and nonrandomized studies. Journal of Educational Psychology.

[CR35] Ruhm CJ (2000). Are recessions good for your health?. The Quarterly Journal of Economics.

[CR36] Ruhm CJ (2016). Health effects of economic crises. Health Economics.

[CR37] Said SE, Dickey DA (1984). Testing for unit roots in autoregressive-moving average models of unknown order. Biometrika.

[CR38] Salinari G, Benassi F (2022). The long-term effect of the Great Recession on European mortality. Journal of Population Research.

[CR39] Tapia Granados JA (2005). Increasing mortality during the expansions of the US economy, 1900–1996. International Journal of Epidemiology.

[CR40] Tapia Granados JA, Ionides EL (2017). Population health and the economy: Mortality and the Great Recession in Europe. Health Economics.

[CR41] Toffolutti V, Suhrcke M (2014). Assessing the short-term health impact of the Great Recession in the European Union: A cross-country panel analysis. Preventive Medicine.

[CR42] White KM (2002). Longevity advances in high-income countries, 1955–96. Population and Development Review.

[CR43] Wilmoth JW, Boe C, Barbieri M, Crimmins EM, Preston SH, Cohen B (2010). Geographic differences in life expectancy at age 50 in the United States compared with other high-income countries. International differences in mortality at older ages. Dimensions and sources.

